# Affordable Senior Housing in Rural Massachusetts – Gaps and Solutions to Improve Services and Supports for Residents

**DOI:** 10.1093/geroni/igaf122.2818

**Published:** 2025-12-31

**Authors:** Natasha Bryant, Steve Kalter, Robyn Stone, Peter Atkins

**Affiliations:** LeadingAge, Washington, District of Columbia, United States; Acumen Marketing Research, Inc., Groton, Massachusetts, United States; LeadingAge, Washington, District of Columbia, United States; Fallon Health, Worcester, Massachusetts, United States

## Abstract

Fallon Health, a managed care organization offering the Senior Care Options (SCO) program in Massachusetts, received a grant from the Executive Office of Health and Human Services to assess and improve supportive services in affordable senior housing in a rural part of the state. Partnering with Acumen and LeadingAge, the team conducted an evaluation of 11 properties through resident surveys (N = 327) and focus groups with property managers (N = 6) and resident service coordinators (RSCs) (N = 5). Findings revealed that most properties have operated for over 20 years and lack infrastructure suited for aging residents. Common health conditions include hypertension and arthritis, with half of residents at risk for depression and a quarter for loneliness. Social engagement is moderate. Residents most often require housekeeping and transportation assistance. While nearly all properties employ RSCs, their on-site presence is limited to adequately address resident needs. Other key challenges include RSC staffing, inconsistent assessments, and a lack of community-based service providers with whom to partner. Recommendations include increasing RSC presence, conducting periodic needs assessments to address resident needs, and strengthening community partnerships to coordinate service delivery, care integration and transportation. Massachusetts should explore using targeted Medicaid and state funds to build RSC capacity, incentivize health and social service providers to team with housing organizations and expand telehealth services. The Fallon Health Navigator program, embedded in these sites, should be strengthened and serve as a model for other SCOs. The solutions could improve the well-being of affordable housing residents and support their ability to age in place.

